# Salicylic Acid, a Plant Hormone, Suppresses Phytophagous Insect Immune Response by Interrupting HMG-Like DSP1

**DOI:** 10.3389/fphys.2021.744272

**Published:** 2021-10-04

**Authors:** Md. Mahi Imam Mollah, Hyong Woo Choi, Inhwa Yeam, Je Min Lee, Yonggyun Kim

**Affiliations:** ^1^Department of Plant Medicals, Andong National University, Andong, South Korea; ^2^Department of Horticulture and Breeding, Andong National University, Andong, South Korea; ^3^Department of Horticultural Science, Kyungpook National University, Daegu, South Korea

**Keywords:** salicylic acid, eicosanoid, HMGB1, immunity, insect

## Abstract

Salicylic acid is a plant hormone that can mediate various plant physiological processes. Salicylic acid can bind to human high mobility group box 1 (HMGB1) and interrupt its role in mediating immune responses. Dorsal switch protein 1 (DSP1) is an insect homolog of HMGB1. In this study, a DSP1 (*Se-DSP1*) encoded in *Spodoptera exigua*, a phytophagous insect, was characterized, and its potential role in immune response was explored. Upon bacterial challenge, Se-DSP1 was localized in the nucleus and released into the hemolymph. The released Se-DSP1 could mediate both cellular and humoral immune responses by activating eicosanoid biosynthesis. Salicylic acid could bind to Se-DSP1 with a high affinity. The immune responses of *S. exigua* were significantly interrupted by SA feeding. Larvae reared on tomatoes with high endogenous SA levels became more susceptible to entomopathogens. Taken together, these results suggest a tritrophic defensive role of plant SA against phytophagous insects.

## Introduction

High-mobility group proteins are non-histone DNA-binding proteins in eukaryotes. These high-mobility group (HMG) proteins include at least three unrelated protein groups: HMGA, HMGB, and HMGN (Reeves, 2003). High-mobility group A proteins bind to A/T-rich DNA sequences and promote the binding of other transcription factors, while HMGN proteins bind to nucleosomes and promote chromatin decondensation (Bustin, 2001). On the other hand, HMGB proteins are characterized by a DNA-binding box with an acidic tail that can non-specifically bind to DNA and induce sharp bends, allowing the easy accession of transcription factors and chromatin remodeling complexes (Thomas and Travers, 2001). In mammals, four HMGB proteins (HMGB1–4) are known. They are ubiquitously expressed during embryogenesis, although they show different spatial expression patterns in the adult stage (Stros, 2010).

Human high mobility group box 1 is a ubiquitously expressed and highly conserved nuclear protein that plays important role in the chromatin organization and transcriptional regulation in mammals (Bianchi et al., 2017). It was first discovered in the calf thymus as a chromatin-associated protein with high acidic and basic amino acid contents (Goodwin and Johns, 1977). Furthermore, HMGB1 consists of two HMG boxes (boxes A and B) and a long acidic tail (Stros et al., 2007). The two HMG boxes have different functions and properties: box A recognizes pre-bent and linear DNA (Muller et al., 2001), while box B binds to mini-circular DNA or bending linear DNA (Webb et al., 2001). Additional motifs have been found in HMGB1. For example, a coiled coil region can mediate protein–protein interactions for the transcriptional control of target genes through nuclear receptors (Parry et al., 2008). A low complexity region contains repeats of single or short amino acid repeat motifs. It plays various functional roles in modulating protein–protein interactions, protein–nucleic acid interactions, and protein subcellular localization (Salichs et al., 2009).

High mobility group box 1 can be released under stress either passively from dead cells (Bianchi et al., 2017) or actively secreted from activated immune cells, enterocytes, hepatocytes, and, possibly, several other cellular types (Tsung et al., 2007). Released HMGB1 can act as a damage-associated molecular pattern (DAMP) and activate the innate immune system by interacting with pattern recognition receptors (Jong and Dong, 2018).

The dorsal switch protein 1 of *Drosophila melanogaster* is a homolog of HMGB1 first identified in insects (Mosrin-Huaman et al., 1998). In addition to HMGB1 signature motifs, dorsal switch protein 1 (DSP1) possesses two glutamine-rich domains at its N-terminus (Canaple et al., 1997). The null mutants of DSP1 exhibit homeotic transformations associated with the downregulation of *Sex combs-reduced* expression (Decoville et al., 2001). Furthermore, DSP1 acts as a transcription factor and a chromatin remodeling factor by enhancing other transcriptional factors such as the Hox protein (Zappavigna et al., 1996), p53 (Jayaraman et al., 1998), and steroid hormone receptor (Boonyaratanakornkit et al., 1998). Another HMGB1 homolog has been found in a mosquito, *Aedes aegypti*. It shows high homologies with HMGB1 and *Drosophila* DSP1 (Ribeiro et al., 2012). This mosquito HMGB1 can facilitate the transcriptional factor Rel to bind to the nuclear factor kappa B (NF-kB) promoter to express antiviral genes against a dengue viral infection (de Mendonça Amarante et al., 2017). In addition to dipteran insects, a lepidopteran insect (*Plodia interpunctella*) also possesses HMGB1-like proteins (Aleporou-Marinou et al., 2003). Thus, HMGB1 might be highly conserved in insects as in mammals and play crucial roles in the immune responses of insects by contributing to chromatin organization and transcriptional regulation. However, little is known about the role of HMGB1 as a DAMP and in subsequent immunity-related responses in insects.

Salicylic acid is a plant hormone that can mediate plant defense against microbial pathogens. It can also regulate plant growth and development (Koo et al., 2020). In particular, the salicylic acid (SA) signal is linked to a resistance to biotrophic microbial pathogens by inducing the expression of pathogenesis-related genes that encode antimicrobial or other defensive proteins and by upregulating reactive oxygen species (Ton et al., 2006). With respect to insect resistance, plants can use the SA signal against phloem-feeding insects, although the efficacy of SA is controversial (Zarate et al., 2007). Little information exists about the SA signal in interactions between plants and chewing insects such as lepidopterans (Wu and Baldwin, 2009). Meanwhile, HMGB1 can bind to SA and lose its proinflammatory activity (Choi et al., 2015). The fact that SA is a metabolite of aspirin (= acetylated SA) in humans suggests a novel non-steroidal anti-inflammatory drug (NSAID) action of aspirin, which is known to inhibit cyclooxygenase 2 (COX-2) to shutdown inflammatory prostaglandin (PG) production (Choi et al., 2015). Cyclooxygenase inhibitors such as aspirin are effective in inhibiting insect immune responses (Stanley and Kim, 2011). We hypothesized that plant SA could participate in plant defense against phytophagous insects by interacting with insect HMGB1 homologs. Thus, it might lead to the suppression of insect immune responses against microbial entomopathogens. Thus, the objective of this study was to perform a functional characterization of DSP1 (Se-DSP1) in the lepidopteran insect *Spodoptera exigua* and subsequent analyses of Se-DSP1 to determine its functional association with plant SA. Our results propose a novel plant defense mechanism utilizing SA against insect pests so that such insects could be susceptible to microbial entomopathogens by interfering with their immune systems.

## Materials and Methods

### Insect Rearing and Bacteria Culture

The larvae of *S. exigua* were reared using the method of Goh et al. (1990) at 25 ± 1°C, with which the larvae underwent five instars (“L1–L5”). For the immune challenge, *Escherichia coli* Top10 (Invitrogen, Carlsbad, CA, United States) were cultured with the method described previously (Hasan et al., 2019). Bacterial cells were resuspended in distilled water to have 4.5 × 10^4^ cells/μl for treatment. For a pathogenicity test, *Bacillus thuringiensis* subsp. *aizawai* (Bt) and *Xenorhabdus hominickii* ANU101 (Xh) were cultured with the method described previously (Mollah et al., 2020a).

### Chemicals

An anticoagulant buffer (ACB) was prepared with 186 mM of NaCl, 17 mM of Na_2_EDTA, and 41 of mM citric acid, and then the pH was adjusted to 4.5 with HCl. Phosphate-buffered saline (PBS) was prepared with 100 mM of phosphoric acid, and the pH was adjusted to 7.4. The transfection reagent Metafectene Pro was purchased from Biontex (Plannegg, Germany). Salicylic acid (2-hydroxybenzoic acid: SA, ≥99%) was obtained from Sigma-Aldrich (Seoul, Korea) and dissolved in dimethylsulfoxide.

### Bioinformatics and Sequence Analysis

A sequence (Se-DSP1) of *S. exigua* homologous to human HMGB1 (GenBank accession number: CAG33144.1) was obtained from a transcriptome (GenBank accession number: GAFU01017850.1). Its open reading frame (ORF) was deposited at GenBank (accession number: MK737894). Phylogenetic and domain analyses were performed using the MEGA6 and Clustal W programs from the European Molecular Biology Laboratory's European Bioinformatics Institute (EMBL-EBI) (www.ebi.ac.uk). Bootstrapping values were obtained with 1,000 repetitions to support branches. Protein domains were predicted using the SMART (http://smart.embl-heidelberg.de/) search program.

### RNA Extraction and RT-qPCR

Total RNA extraction, complementary DNA (cDNA) preparation, and quantitative reverse transcription (RT-q)PCR were performed following the methods described previously (Mollah et al., 2020a,b). The synthesized first-stranded cDNA was used as a template for PCR amplification with 35 cycles of 94°C for 30 s, 58.8°C for 30 s, and 72°C for 30 s after an initial heat treatment at 94°C for 2 min with the gene-specific primers listed in Supplementary Table S2.

### RNA Interference (RNAi) of *Se-DSP1* Expression

The DNA template was amplified with the gene-specific primers of Se-DSP1 (Supplementary Table S2) containing a T7 promoter sequence (5′-TAATACGACTCACTATAGGGAGA-3′) at the 5′ end. Double-stranded RNA (dsRNA) was prepared following the method described previously (Mollah et al., 2020b). A viral gene, *CpBV302*, was used as a control dsRNA (“dsCON”).

### Analyses of Immune Responses

For a cellular immune assay, hemocytic nodules were assessed with the method described previously (Mollah et al., 2020a). For humoral immune response, the expression levels of six antimicrobial peptide (AMP) genes (Supplementary Table S2) were assessed with the RT-qPCR method as described above.

### Purification of Recombinant *Se-DSP1* and SA Binding Affinity

The ORF of Se-DSP1 was cloned into a pET expression vector (Invitrogen, Carlsbad, CA, United States). After overexpression with isopropyl β-D-thiogalactopyranoside, the recombinant protein containing a 6× His tag was purified with the method described previously (Mollah et al., 2021). Resulting proteins contained partial degraded fragments, which were removed by gel filtration chromatography with Sephadex G-100 resin (Sigma-Aldrich, Seoul, Korea). To detect the binding of Se-DSP1 to SA, a thermal shift assay (Simeonov, 2013) was performed with a Protein Thermal Shift (Applied Biosystem, Foster City, CA, United States) dye kit according to the instructions of the manufacturer. Briefly, a reaction mixture consisted of 5 μl of a protein thermal shift buffer, 2.5 μl of protein thermal shift dye (8×, prepared by kit), 10 μl (500 ng) of purified protein (Se-DSP1), and 2.5 μl of ligands at different final concentrations (0, 5, 10, 15, and 20 μM). A melting curve experiment type was set up using the StepOne Real Time PCR System (Applied Biosystems, Foster City, CA, United States) with a continuous ramping mode (2.96°C/min), at which two thermal extremes were 25°C for 2 min at the initial step and 99°C for 2 min at the final step. Melting temperatures resulting from the experiment were plotted with SigmaPlot 10.0 (Systat Software, San Jose, CA, United States). A dissociation constant (Kd) was calculated using the ligand binding equation category.

### Phospholipase A_2_ (PLA_2_) Enzyme Activity, Western Blotting, and Immunofluorescence Staining

Phospholipase A2 enzyme activity was measured with a PLA_2_ Assay Kit (Cayman Chemical, Ann Arbor, MI, United States) using diheptanoyl thio-phosphatidyl choline for the secretory phospholipase A2 (sPLA_2_) substrate and arachidonyl thio-phosphatidyl choline for the cellular PLA_2_ (cPLA_2_) substrate after sample preparation as described previously (Vatanparast et al., 2018). Briefly, the L5 larvae of *S. exigua* were injected with dsDSP1 and dsCON (1 μg/larva) using a microsyringe (Hamilton, Reno, NV, United States), incubated at room temperature for 24 h, and then challenged with *E. coli* (10^4^ cells/larva). At 8 h post injection of the bacteria, fat body was collected. The reaction volume consisted of 10 μl of fat body extract, 10 μl of 5,5-dithio-bis-(2-nitrobenzoic acid), 5 μl of the assay buffer, and 200 μl of the substrate. The change in absorbance at 405 nm was monitored with three biologically independent replications.

For the Western blot, hemolymph samples were collected from the L5 larvae of *S. exigua*. After centrifugation with 800 × g for 3 min at 4°C, the supernatant plasma samples were obtained and used for the analysis. A polyclonal antibody against rat HMGB1 (NB100-2322; Abcam, Waltham, MA, United States) was used after 5,000 times of dilution. α-Tubulin was used as a reference protein and detected by a polyclonal antibody (GTX112141; GeneTex, Irvine, CA, United States) after 1,000 times of dilution. The primary antibodies were detected with an anti-rabbit immunoglobulin G (IgG)–alkaline phosphatase secondary antibody (WP20007; Sigma-Aldrich, Seoul, Korea) after incubation with a substrate (BICP/NBT; Sigma-Aldrich, Seoul, Korea). This antibody showed a specific cross-reactivity with Se-DSP1 (Supplementary Figure S1).

An immunofluorescence assay (IFA) was performed according to the method described previously (Mollah et al., 2021). Briefly, hemocytes and fat body tissues were collected from the L5 larvae of *S. exigua*. Furthermore, Se-DSP1 was observed with the HMGB1 antibody. The cytoplasm and nucleus were stained with Alexa Fluor 555 phalloidin and 4′,6-diamidino-2-phenylindole (DAPI, 1 μg/ml) (Thermo Fisher Scientific, Rockford, IL, United States), respectively. Subsequently, cells were observed under a fluorescence microscope (DM2500; Leica, Wetzlar, Germany) at 400× magnification under microscope parameter conditions (Supplementary Table S3).

### Bioassay for Bacterial Pathogens

To determine Bt virulence, the L4 larvae were subjected to feeding assays using a leaf-dipping method. Briefly, a piece of cabbage leaf (3 × 3 cm) was soaked in 250 ppm of a Bt suspension containing different concentrations of SA for 5 min. The treated leaves were then provided to larvae to allow feeding for 24 h. To determine Xh virulence, the L4 larvae were subjected to injecting assays. Each larva was injected with 100 live bacterial cells containing different concentrations of SA using a microsyringe (Hamilton, Reno, NV, United States). The treated larvae were then incubated for another 4 days under rearing conditions, and mortality was monitored. Each replication consisted of 10 larvae. Each treatment was replicated three times.

### Effect of Tomato Plants on Susceptibility of *S. exigua* to Bacterial Pathogens

Two lines of *Solanum esculantum*, “M82” and “IL2-2,” were used. Seeds were obtained from the Tomato Genetics Resource Center at the University of California (Davis CA, United States) (http://tgrc.ucdavis.edu/). For the bioassays, the L2 larvae of *S. exigua* were reared on 4-week-old tomato plants for 7 days with a 16-h light/8-h dark period at a temperature of 25 ± 2°C. The larvae were then used to assess immune responses indicated by nodule formation and AMP expression, as described above. The larvae were also used to determine bacterial virulence, as described above.

### Quantification of SA in Tomato Leaves

Approximately 300 mg of leaf tissues were collected from each line of tomato plants, and then extracted and analyzed for SA levels, as described previously (Bowling et al., 1994). The SA concentration was determined by high-performance liquid chromatography (HPLC) with an ARH-601 organic acid column (100 × 6.5 mm; Transgenomic Inc., Omaha, NE, United States) run at 45°C in.01 N of H_2_SO_4_ and a flow rate of 0.6 ml/min.

## Results

### Prediction of a DSP1 Homolog *(Se-DSP1)* From *S. exigua*

By interrogation with a *D. melanogaster* DSP1 sequence (GenBank accession number: NP_727960.1) as a query, *Se-DSP1* was predicted from a transcriptome (GenBank accession number: GAFU 01017850.1). Its ORF consisted of 1,518 bp encoding 505 amino acids. *Se-DSP1* shared 95.1, 77.1, 73, and 52.1% amino acid sequence similarities with the DSP genes of *Helicoverpa armigera, Leptinotarsa decemlineata, D. melanogaster*, and the HMGB1 of *Homo sapiens*, respectively. A phylogenetic analysis showed that *Se-DSP1* was closely related to other lepidopterans within an insect cluster (Supplementary Figure S2A). Like mammalian HMGB1, *Se-DSP1* comprised HMG boxes A and B and an acidic tail (Supplementary Figure S2B). Phosphorylation and disulfide linkage sites are frequently located in box A compared with other domains. In the two HMG boxes, human *HMGB1* and *Se-DSP1* exhibited higher sequence identities (69.9%) in box A than in box B (39.4%). Unlike mammalian HMGB1s, insect DSP1s possess additional N-terminal domains such as a coiled coil, low complexity, and RNase.

### Expression Profile of *Se-DSP1*

In all developmental stages, *Se-DSP1* was expressed showing high expression levels at the L5 and adult stages. It had low expression levels at the pupal stage (Supplementary Figure S3A). At the L5 larval stage, *Se-DSP1* was expressed in all the tested tissues such as hemocytes, fat body, gut, and epidermis (Supplementary Figure S3B). The basal expression levels of *Se-DSP1* were significantly upregulated following the immune challenge with *E. coli* in hemocytes and the fat body (Supplementary Figure S3C). Such an upregulation was more acute in hemocytes than in the fat body.

### Translocation of *Se-DSP1* Upon Immune Challenge

**Figure 1:**
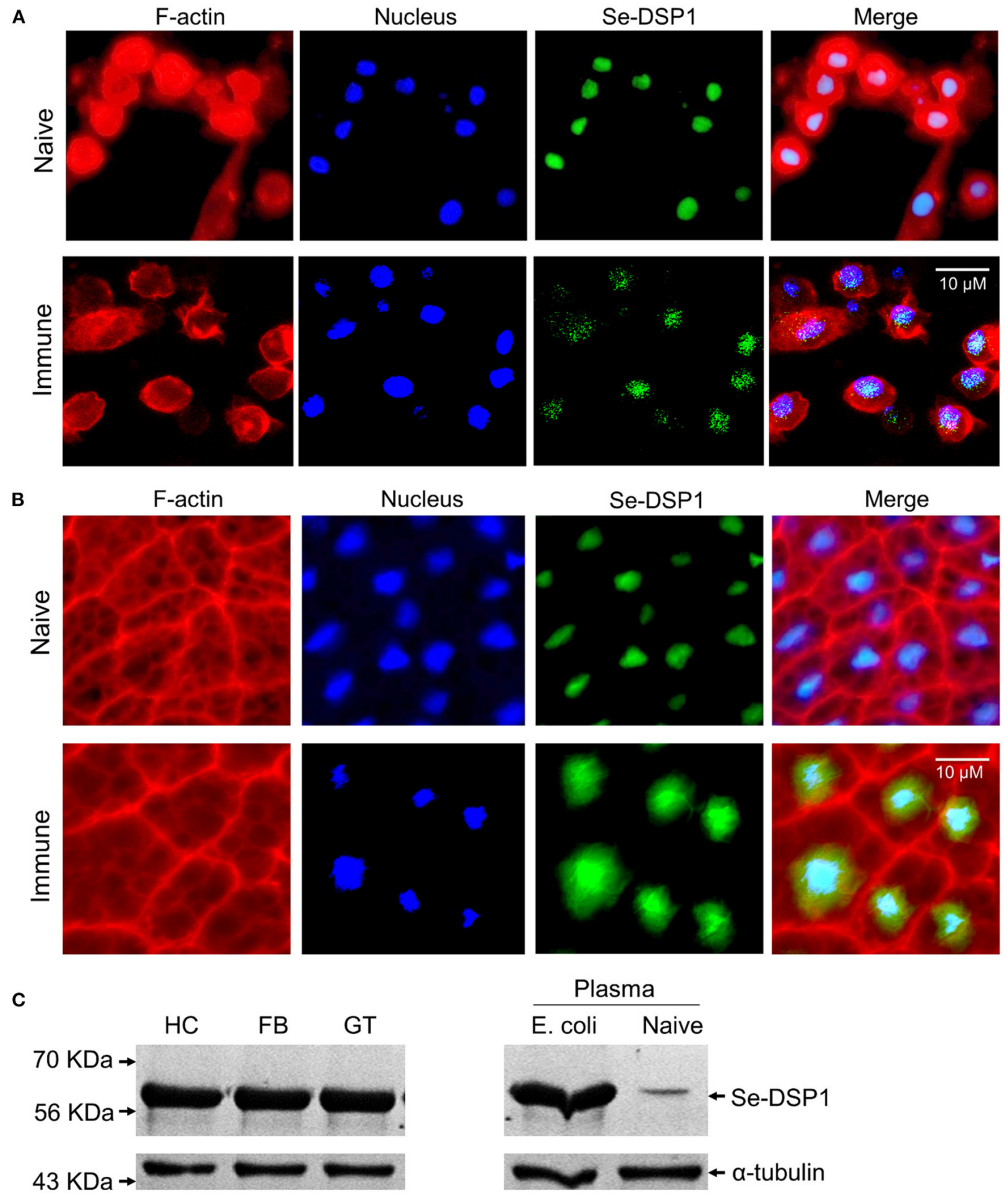
The release of dorsal switch protein 1 (DSP1) *Spodoptera exigua* (Se-DSP1) proteins from nuclei in response to an immune challenge. **(A)** The mobilization of Se-DSP1 in hemocytes. **(B)** The mobilization of Se-DSP1 in the fat body. Cytosol and nuclei were stained with phalloidin against F-actin and 4′,6-diamidino-2-phenylindole (DAPI) against nuclear DNA. Se-DSP1 was detected with a polyclonal antibody raised against mammalian high mobility group box 1 (HMGB1). Supplementary Table S3 describes the setting values of the fluorescence microscope to get the immunofluorescence images. **(C)** Western blotting against different tissue proteins of hemocyte (“HC”), fat body (“FB”), and midgut (“GT”). For the immune challenge, each of the L5 larvae were injected with 4.5 × 10^4^ heat-killed (HK) *Escherichia coli* cells. Plasma samples were collected 6 h post injection (PI). A homogenous immunoblotting intensity against α-tubulin indicates the equal amount of protein sample loading in each lane.

In naïve larvae, Se-DSP1 was localized in the nuclei of the hemocytes (Figure 1A). Upon bacterial challenge, some Se-DSP1 proteins migrated to the cytosol. Similarly, Se-DSP1 was localized in nuclei of the fat body and isolated from naïve larvae, although some of them were detected in the cytosol after the immune challenge (Figure 1B). Interestingly, Se-DSP1 was detected in the plasma of the immune-challenged larvae, although it was slightly detected in the plasma collected from the naïve larvae (Figure 1C).

### RNAi of *Se-DSP1* Expression Results in Immunosuppression

**Figure 2:**
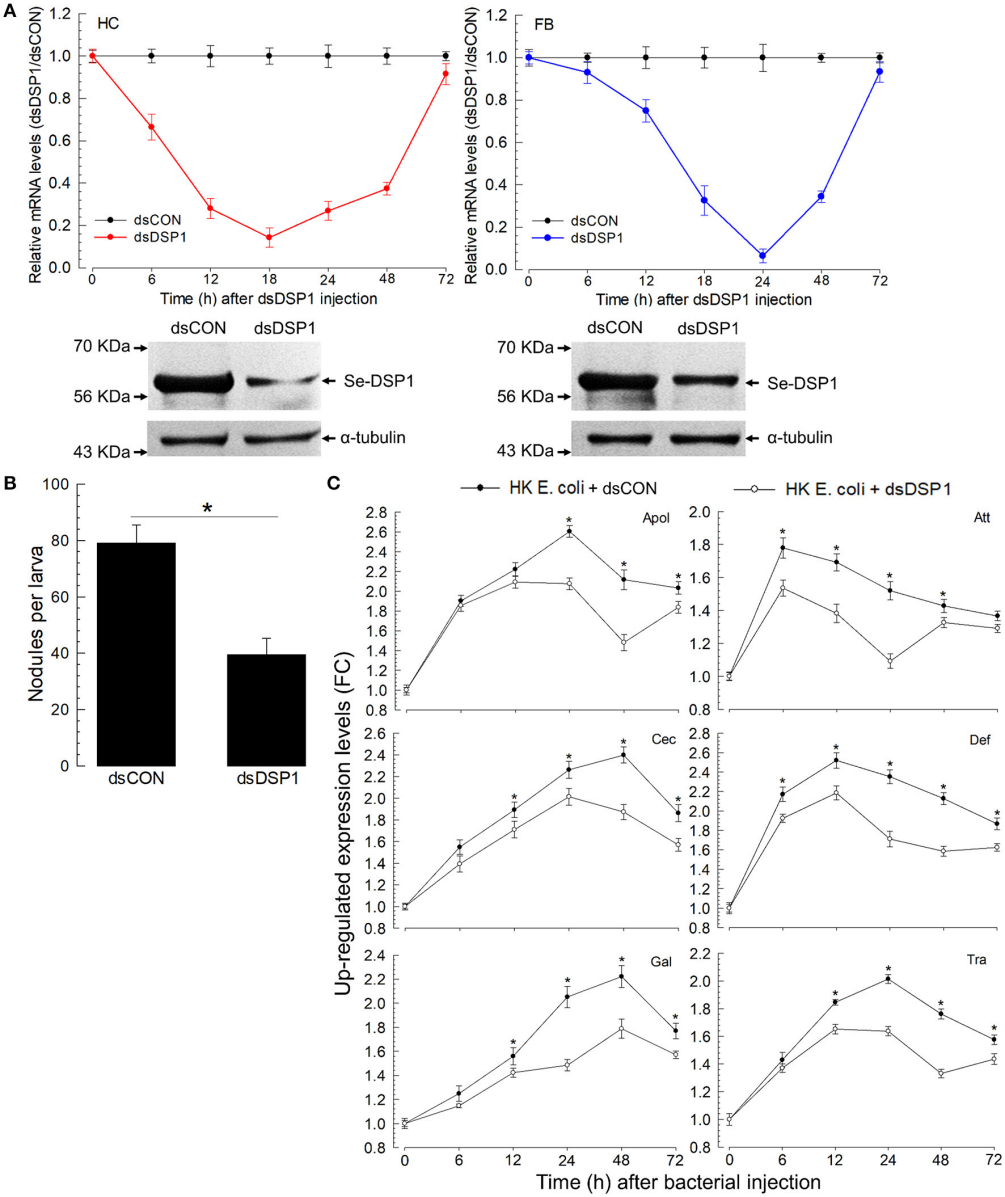
Effects of ribonucleic acid interference (RNAi) on *Se-DSP1* expression and immune responses of *S. exigua*. **(A)** The temporal change of Se-DSP1 expression level after the treatment of L5 larvae with double-stranded (ds)RNA specific to Se-DSP1 (1 μg per larva). A viral gene, *CpBV302*, was used as a control dsRNA (“dsCON”). The lower panels indicate Western blotting against HC and FB extracts. A homogenous immunoblotting intensity against α-tubulin indicates the equal amount of protein sample loading in each lane. **(B)** The suppression of hemocyte nodule formation. For the bacterial challenge, HK *E. coli* (4.5 × 10^4^ cells per larva) were injected into each larva 24 h after dsRNA treatment. At 8 h PI, the number of nodules was assessed. **(C)** The suppression of six AMP gene expressions after RNAi treatment. A ribosomal protein, RL32, was used as an internal control. Relative expression levels were determined by normalization compared with the levels of the control gene expression followed by the calculation of relative ratios compared with the lowest level. Each treatment was replicated three times. Asterisks above SD bars indicate a significant difference among means at Type I error = 0.05 (least significant difference, LSD, test).

To analyze the role of *Se-DSP1* in association with immune responses, its expression was manipulated by RNAi with gene-specific dsRNA (Figure 2A). Such an RNAi treatment significantly reduced *Se-DSP1* expression levels by more than 90% in the hemocytes and fat body at 24 h. The reduced mRNA levels were confirmed by weaker bands in Western blotting against Se-DSP1. At such a reduced expression level of *DSP1*, cellular immune response was measured by counting hemocytic nodules (= a cellular immune response sequestering small pathogens with hemocytes and subsequent melanization) after the bacterial challenge (Figure 2B). The RNAi-treated larvae exhibited a significant (*P* < 0.05) impairment in cellular immune response based on nodule formation. Furthermore, the bacterial challenge upregulated the gene expression levels of six AMPs in the control larvae (Figure 2C). The reduction of AMP gene expression in the RNAi-treated larvae was significant (*F* = 997; df = 1, 144; *P* < 0.0001) compared with that in the control larvae.

### *Se-DSP1* Activates Eicosanoid Biosynthesis to Activate Immune Responses

**Figure 3:**
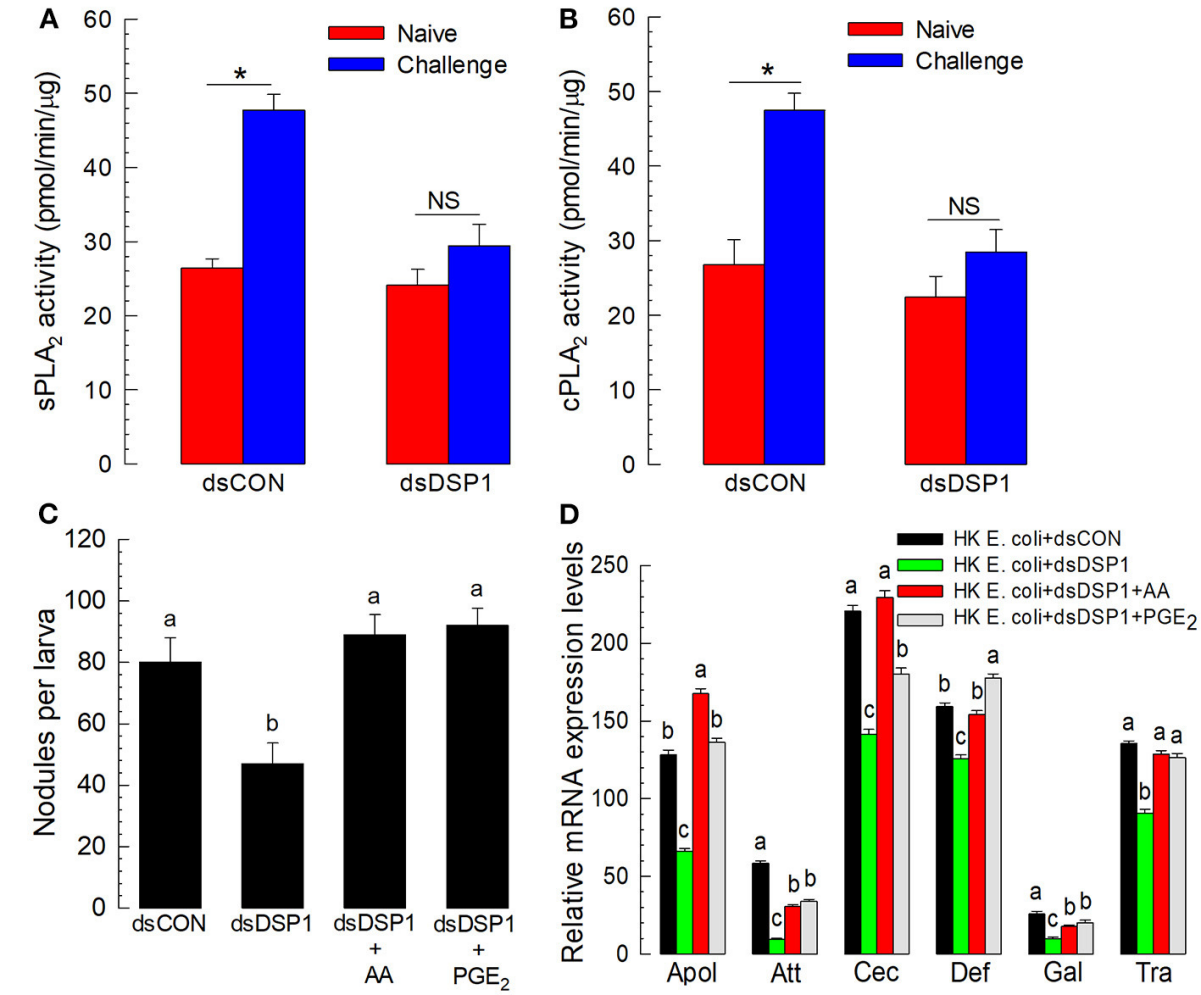
Se-DSP1 activates eicosanoid biosynthesis to mediate the cellular and humoral immune responses of *S. exigua*. The suppression of bacteria-induced **(A)** secretory phospholipase A2 (sPLA_2_) and **(B)** cellular PLA2 (cPLA_2_) activities by RNAi specific to *Se-DSP1* expression. A viral gene, *CpBV302*, was used as a control dsRNA (“dsCON”). For the immune challenge, each of the L5 larvae was injected with 4.5 × 10^4^ HK *E. coli* cells 24 h after an injection with dsRNA specific to *Se-DSP1* (dsDSP1) (1 μg per larva). At 8 h after the immune challenge, fat bodies were collected and used for enzyme assays. An asterisk on SD bars indicates a significant difference among means at Type I error = 0.05 (LSD test). “NS” stands for no significant difference. **(C)** the rescue effect of eicosanoids on immune responses suppressed by RNAi treatment in hemocytic nodule formation. **(D)** Antimicrobial peptide (AMP) gene expression. Arachidonic acid (AA) or prostaglandin E_2_ (PGE_2)_ at a dose of 10 μg per larva was injected along with HK *E. coli* (4.5 × 10^4^ cells per larva) 24 h post dsRNA injection,. Nodule formation was assessed, and fat body tissues were collected and used to analyze AMP gene expression 8 h after *E. coli* injection. A ribosomal protein, *RL32*, was used as an internal control. Each treatment was replicated three times. Different letters above standard deviation bars indicate significant difference among means at Type I error = 0.05 (LSD test).

Eicosanoids mediate various insect immune responses (Stanley and Kim, 2011). To understand the immune mediation of Se-DSP1, its activation of eicosanoid immune signaling was tested by measuring PLA_2_ activities, catalyzing the committed step for eicosanoid biosynthesis, after RNAi treatment against *Se-DSP1* (Figure 3). Two substrates, arachidonate phospholipid and non-arachidonate phospholipid, were used to measure PLA_2_ enzyme activities. In particular, the PLA_2_ activity obtained from the non-arachidonate phospholipid was designated as sPLA_2_ activity. In contrast, cPLA_2_ activity was obtained from PLA_2_ activity using the arachidonate phospholipid. The immune challenge significantly (*P* < 0.05) increased both PLA_2_ activities (Figures 3A,B). However, the larvae treated with RNAi specific to *Se-DSP1* expression failed to induce PLA_2_ activities. Such suppressed immune responses by RNAi treatment were significantly (*P* < 0.05) rescued by arachidonic acid (AA) or prostaglandin E_2_ (PGE_2_) in cellular (Figure 3C) or humoral immunity (Figure 3D).

### *Se-DSP1* Alone Induces PLA_2_ Activity

**Figure 4:**
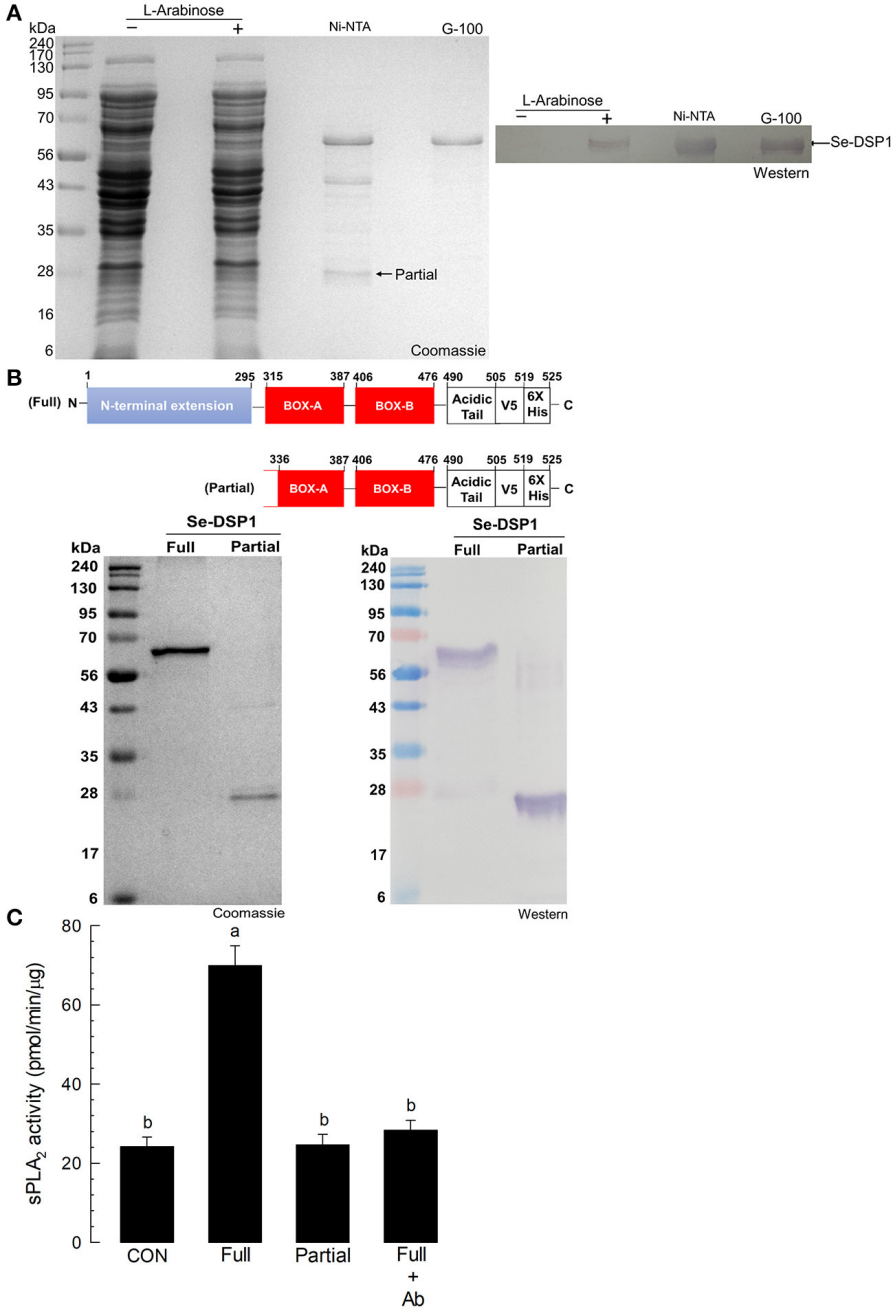
The activation of fat body PLA_2_ enzyme activity by Se-DSP1. **(A)** The purification of recombinant Se-DSP1 by affinity chromatography (“Ni-NTA”) and size-exclusion chromatography (“G-100”). The purified protein was confirmed with a monoclonal antibody specific to the V5 tag of the recombinant Se-DSP1 by Western blotting. **(B)** the purification of partial Se-DSP1. A comparison of protein domains between full and partial Se-DSP1. The partial protein was confirmed in its sequences by liquid chromatography with tandem mass spectrometry (LC-MS/MS) (Supplementary Figure S4). **(C)** The upregulation of the sPLA_2_ activity of *S. exigua* larvae by injecting a full length of Se-DSP1 (1 μg/larva). The enzyme activity in the fat body was measured 8 h after the injection. Partial Se-DSP1 was injected in the same amount. Control (“CON”) represented the phosphate-buffered saline (PBS) injection. An antibody (“Ab”) addition (2 μl/larva) used a commercial polyclonal antibody that recognized Se-DSP1. Each treatment was replicated three times. Different letters above SD bars indicate a significant difference among means at Type I error = 0.05 (LSD test).

A recombinant protein of Se-DSP1 was generated using a bacterial expression system. The recombinant protein was then purified using an affinity chromatography system containing nickel-nitrilotriacetic acid (Ni-NTA) resin and further cleared with a gel filtration chromatography system containing Sephadex G-100 (Sigma-Aldrich, Seoul, Korea) (Figure 4A). During the purification of the full-length Se-DSP1, a partial protein was also purified and confirmed by a Western analysis against a V5 epitope (Figure 4B). The truncated protein was assessed by liquid chromatography with tandem mass spectrometry (LC-MS/MS) and showed its partial structure deleting the N-terminal extension domain (Supplementary Figure S4). The injection of Se-DSP1 significantly increased the PLA_2_ activity of the naïve larvae (Figure 4C). However, the partial Se-DSP1 failed to induce enzyme activity. The enzyme induction with full-length Se-DSP1 was prevented by the addition of an HMGB1 antibody recognizing Se-DSP1 (Figure 4C).

### Salicylic Acid (SA), a Plant Hormone, Antagonizes Mediating Effects of *Se-DSP1* in Insect Immune Responses

**Figure 5:**
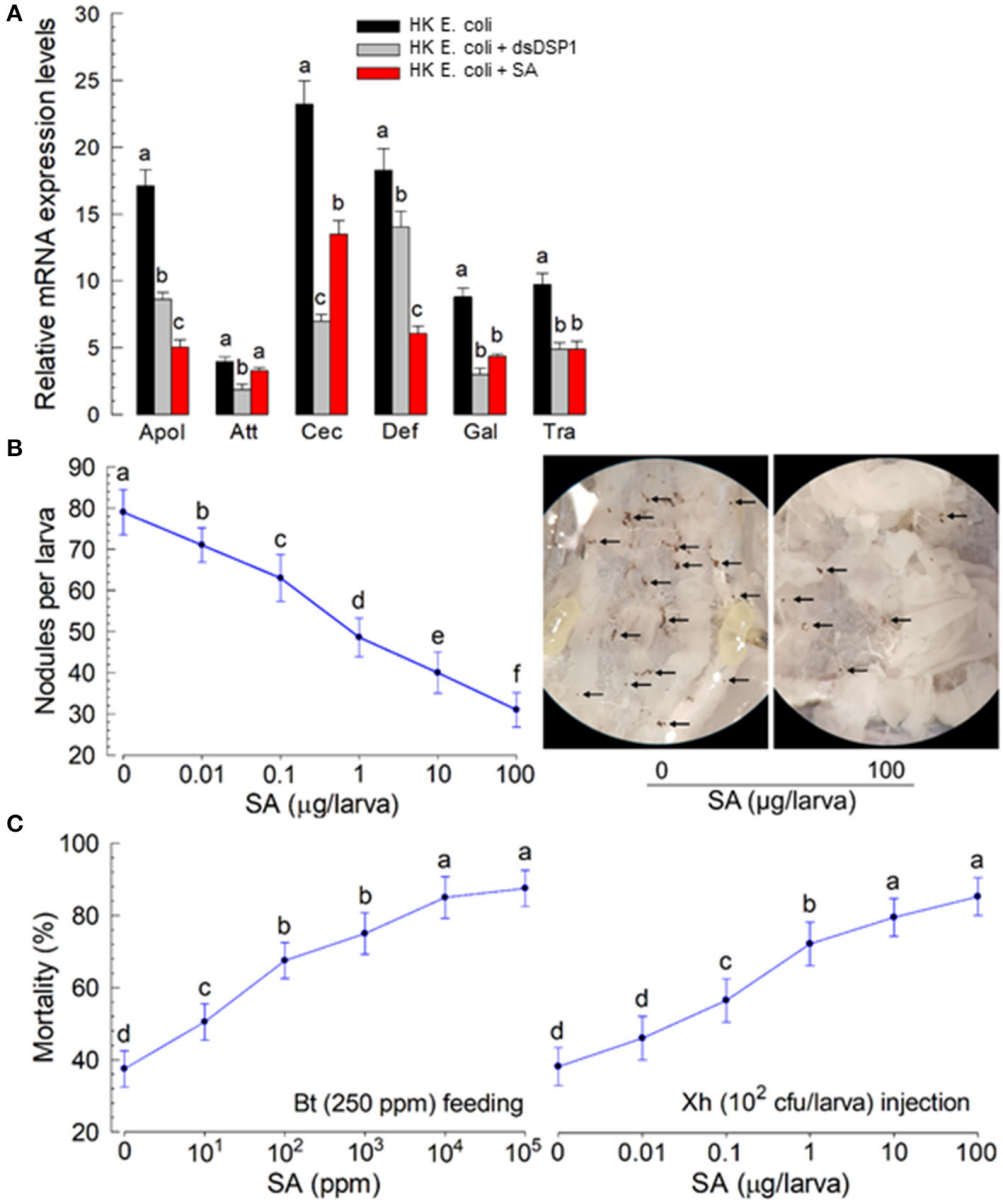
Insect immunosuppression induced by plant hormone salicylic acid (SA) in *S. exigua*. **(A)** The suppression of AMP gene expression by SA in response to bacterial infection. The effect of SA was compared to that of RNAi specific to *Se-DSP1* expression. The RNAi treatment was performed by injecting dsRNA specific to *Se-DSP1* (dsDSP1) (1 μg per larva). SA was injected to the L5 larvae at a dose of 10 μg per larva along with bacterial injection, 24 h after a dsDSP1 injection. For the bacterial challenge, HK *E. coli* (5.5 × 10^4^ cells per larva) were injected into the larvae. After 6 h, fat bodies were collected and used to assess the expression levels of six AMP genes: apolipophorin III (“Apol”), attacin I (“Att”), cecropin (“Cec”), defensin I (“Def”), gallerimycin (“Gal”), and transferrin I (“Tra”). A ribosomal protein, *RL32*, was used as an internal control. Each treatment was replicated three times. A bacterial challenge to RNAi-treated larvae was performed 24 h after a dsDSP1 injection. **(B)** The suppression of cellular immune response by SA treatment. Hemocyte nodule formation was assessed by injecting HK *E. coli* at 4.5 × 10^4^ cells per larva. Nodules (see photos taken at ×50 magnification) were counted 8 h after an *E. coli* injection. **(C)** Enhanced bacterial pathogenicity by SA treatment. The pathogenic test used two bacterial pathogens: *Bacillus thuringiensis* subsp. *aizawai* (Bt) by feeding and *Xenorhabdus hominickii* (Xh) by injection. Each treatment was replicated three times. For bioassays, 10 larvae were used per replication. Different letters above SD bars indicate a significant difference among means at Type 1 error = 0.05 (LSD test).

Salicylic acid is known to bind to human HMGB1 and interrupt immune mediation (Choi et al., 2015). This suggests that it might antagonize the immune-mediating role of Se-DSP1, which is an insect homolog of human HMGB1. To test this hypothesis, different doses of SA were injected to larvae. The immune responses of the larvae were then assessed (Figure 5). Six AMP genes expressed in response to the bacterial challenge exhibited significant (*P* < 0.05) reductions in their expression levels after RNAi treatment using dsRNA specifically targeting the *Se-DSP1* expression (Figure 5A). Among the six AMPs regulated by Se-DPS1, SA significantly (*P* < 0.05) prevented the upregulation of the expression of the AMP genes, except for attacin 1 (*Att*).

The immunosuppressive role of SA in immune responses was supported further by a mixture treatment of SA and entomopathogens. Nodule formation in response to the bacterial (*X. hominickii*) challenge was suppressed by SA injection in a dose-dependent manner (Figure 5B). The addition of SA to *X. hominickii* also enhanced bacterial pathogenicity by a hemocoelic injection assay (Figure 5C). The suppressive effect of SA was also observed after SA-feeding treatment. The addition of SA to *Bacillus thuringiensis* treatment significantly enhanced the bacterial pathogenicity after oral administration.

**Figure 6:**
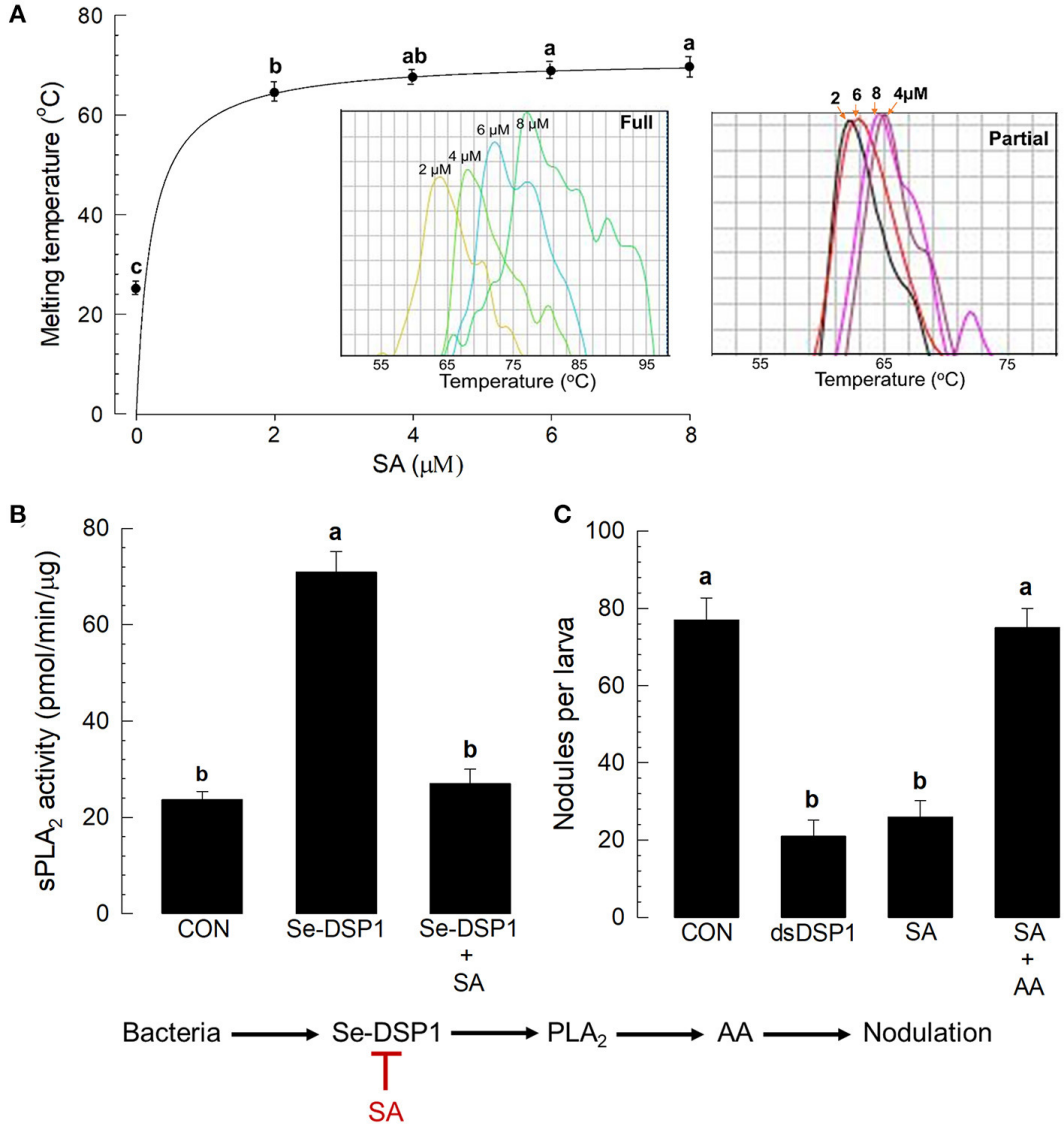
Salicylic acid binding to Se-DSP1 and the suppression of immune mediation. **(A)** The binding affinity of SA to Se-DSP1 by a thermal shifting assay (inset figures of full and partial Se-DSP1). **(B)** The suppression of sPLA_2_ activity induced by Se-DSP1 (1 μg/larva). The enzyme activity in the fat body was measured 8 h after the injection. SA was injected to the L5 larvae at a dose of 10 μg per larva. Control (“CON”) represented the PBS injection. **(C)** The rescue of SA-suppressed nodulation by the addition of AA (10 μg per larva). For the nodulation assay, HK *E. coli* were injected at a dose of 4.5 × 10^4^ cells per larva. At 8 h after the bacterial injection, nodule formation was assessed. Each treatment was replicated three times. Different letters above SD bars indicate a significant difference among means at Type I error = 0.05 (LSD test).

The immunosuppressive role of SA suggested that it might bind to Se-DSP1 and inhibit its immune mediation. In addition, Se-DSP1-like human HMGB1 (Choi et al., 2015) possesses highly conserved residues Arg and Lys to interact with SA (Supplementary Figure S5). To test the direct binding hypothesis, the purified Se-DSP1 protein was assessed for its binding affinity to SA *via* thermal stability assay (Figure 6A). When the concentration of SA was increased, the melting temperature (median dissociation temperature between SA and Se-DSP1 following denaturation) increased (see inset “full” figure in Figure 6A). In contrast, the partial Se-DSP1 did not show a thermal shift of the melting points in different SA concentrations (see inset “partial” figure in Figure 6A). The thermal shift curve of the full-length Se-DSP1 was used to estimate its dissociation constant (Kd) with SA: 1.28 ±.05 μM. The binding of SA to Se-DSP1 resulted in the inhibition of PLA_2_ induction (Figure 6B). In addition, the inhibition of PLA_2_ activity by SA treatment led to the suppression of cellular immune response assessed by nodulation (Figure 6C). The effect of the SA treatment was similar to that of the RNAi treatment against *Se-DSP1* expression. Because SA inhibited Se-DSP1 and subsequent PLA_2_ activity (see the diagram), the addition of AA significantly (*P* < 0.05) rescued the cellular immune response.

### SA Content in Tomato Plants Increases Insect Susceptibility to Entomopathogens

**Figure 7:**
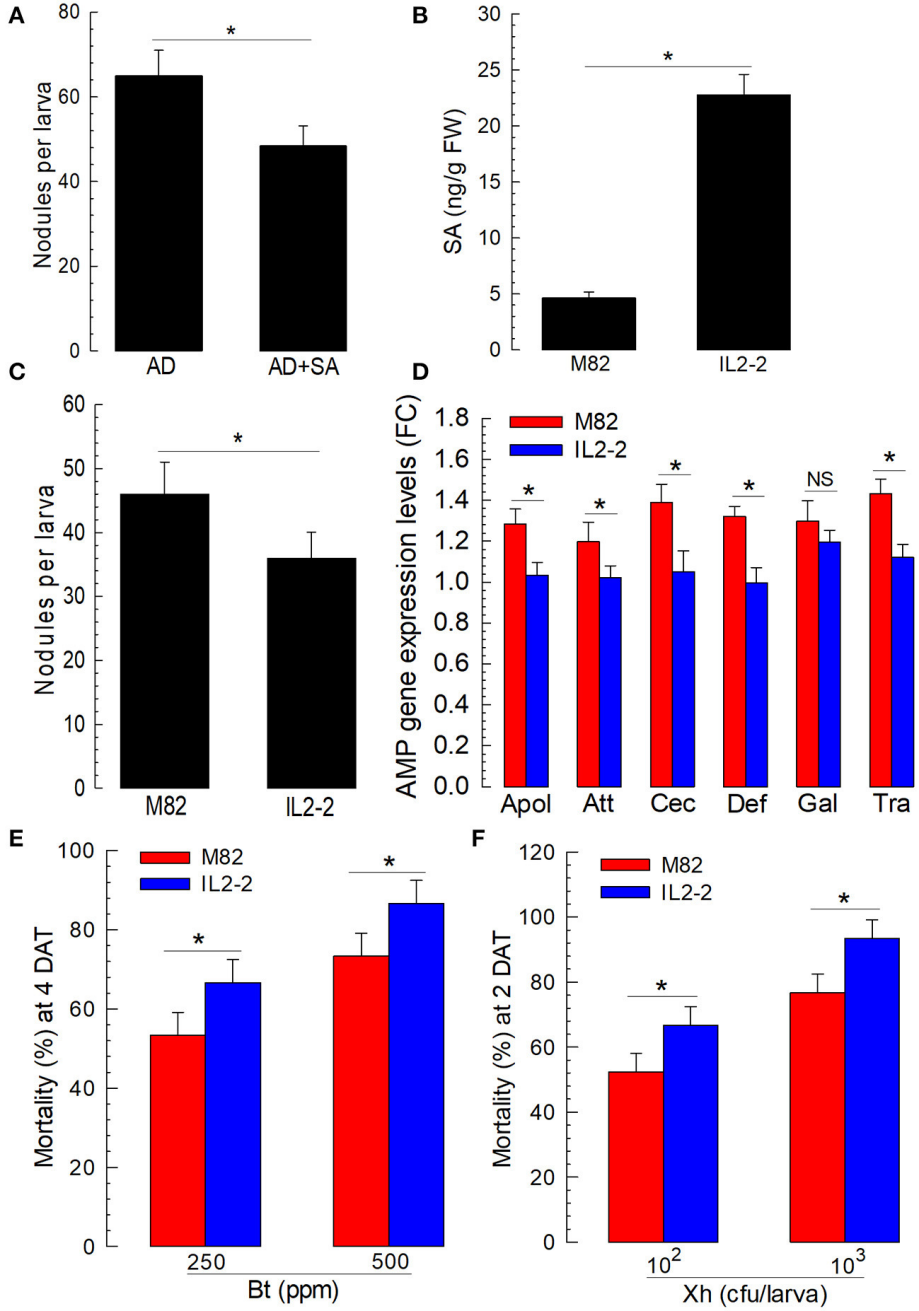
Influence of different tomato varieties on the immune responses and susceptibilities of *S. exigua* larvae to entomopathogens. **(A)** The suppression of cellular immune response by the addition of SA to artificial diet (“AD + SA”). AD was dipped in 1,000 ppm of the SA solution and used to feed L5 larvae for 3 days. The control diet (AD) was treated with dimethyl sulfoxide (DMSO). To assess cellular immune response, hemocyte nodule formation was determined after injecting HK *E. coli* at 4.5 × 10^4^ cells per larva. Nodule formation was assessed 8 h after the *E. coli* injection. **(B)** SA contents in M82 and IL2-2 (*n* = 5). **(C)** The suppression of nodulation with a high SA line, IL2-2. **(D)** The suppression of humoral immune response with a high SA line, IL2-2. For the assessment of humoral immune response, HK *E. coli* (4.5 × 10^4^ cells per larva) were injected. After 6 h, fat bodies were collected and used to assess the expression levels of six AMP genes: apolipophorin III (“Apol”), attacin 1 (“Att”), cecropin (“Cec”), defensin I (“Def”), gallerimycin (“Gal”), and transferrin I (“Tra”). A ribosomal protein, *RL32*, was used as an internal control. Each treatment was replicated three times. **(E,F)** The influence of M82 and IL2-2 on the susceptibility of *S. exigua* larvae to entomopathogens of Bt and Xh. To determine pathogenicity, a feeding assay was performed for Bt, while a hemocoelic injection was performed for Xh. Each treatment was replicated three times. For each replication, 10 L2 larvae were used. Asterisks indicate a significant difference between control and treatment at Type I error = 0.05 (LSD test).

The immunosuppressive effect of SA on insect immunity suggested that plants might use SA to suppress the immune responses of phytophagous insects, leading the insects to be susceptible to entomopathogens. Before we tested the efficacy of SA in a plant host, an artificial diet containing SA was fed to *S. exigua* larvae and showed significant suppression in a cellular immune response (Figure 7A). Then, to test the role of SA in plant defense response against insects, an isogenic tomato line and its parental line were selected because of their different SA contents (Figure 7B). In particular, IL2-2 is an isogenic line generated by introgressing a segment of chromosome 2 from *Solanum pennellii* “LA716” to the *S. lycopersicum* “M82” variety by successive backcrosses (Eshed and Zamir, 1995). Furthermore, IL2-2 possesses a significantly higher amount of SA amount M82.

The larvae of *S. exigua* were fed with tomato leaves. Their immune responses were then assessed after a bacterial challenge. There was a significant (*P* < 0.05) difference in cellular immunity based on nodule formation between the larvae fed with M82 and those fed with IL2-2 (Figure 7C). The expression levels of the six AMP genes regulated by Se-DSP1 were then compared among the larvae fed with two different lines of tomato. The expression levels of the AMPs, except for *gallerimycin*, exhibited a significant (*P* < 0.05) reduction after the bacterial challenge when the larvae were fed with IL2-2 (Figure 7D).

To explore the role of SA in plant defense response by suppressing the insect immune system, the *S. exigua* larvae fed with either M82 or IL2-2 were treated with entomopathogens (Figures 7E,F). Both entomopathogenic bacteria (*B. thuringiensis* and *X. hominickii*) showed significantly different virulence levels against the larvae fed with either M82 or IL2-2. The larvae fed with IL2-2 were more susceptible to both bacterial pathogens compared with larvae fed with M82, indicating that endogenous SA in plants could effectively suppress insect immune defenses to two different entomopathogenic bacteria.

## Discussion

Mammalian HMGB1, a major player in chromatin organization and transcription regulation, can activate innate immune responses by acting as a DAMP. Although HMGB1-like proteins, also known as DSP1s, have been discovered and studied in diverse insects, their contribution to innate immune response has yet to be well-understood. This study characterized the immunological role of Se-DSP1 by acting as a DAMP and its significance in the co-evolutionary context of host plants.

Molecular characterization *via* phylogenetic analysis and domain analysis showed that insect DSP1s, such as Se-DSP1, were different from other HMGB1s. Insect DSP1s were separately clustered in the phylogenetic analysis. They possessed additional unique domains in addition to domains (two HMG boxes and a C-terminal acidic tail) commonly shared by HMGB1. The extended N-terminal tail in DSP1s contains a coiled coil, low complexity, and RNase PH domains. The longest HMGB1-like molecule in this analysis was Bm-DSP1 (625 amino acid residues) compared to human HMGB1 (215 residues). Another difference is the length of the C-terminal acidic tail. Human HMGB1 has a longer acidic tail with 30 residues, compared with the acidic tail of Bm-DSP1 and Se-DSP1, which only has 15 and 18 residues, respectively. Despite structural variations, Dm-DSP1 exhibited similar DNA-binding properties like mammalian HMGB (Zappavigna et al., 1996; Boonyaratanakornkit et al., 1998; Jayaraman et al., 1998). Furthermore, a mutant DSP1 lacking additional N-terminal domains and wild-type Dm-DSP1 have been found to have similar DNA-binding and subsequent bending activities (Janke et al., 2003). This suggests that the additional domains might have physiological functions other than chromatin organization. Our current study showed that the deletion of the N-terminal extension domain led to the impairment of the immune mediation of Se-DSP1. This suggests that the N-terminal domain plays a crucial role in activating PLA_2_ catalytic activity to mediate immune responses in insects.

Upon the immune challenge, *Se-DSP1* expression was highly inducible. This protein was then released into the hemolymph. Furthermore, *Se-DSP1* was expressed in all the developmental stages of *S. exigua* and the four larvae tissues tested in this study. This is because Se-DSP1, like other HMGB1s, might be a non-histone component of chromatin in the nucleus (Andersson and Tracey, 2011). Its basal expression was highly upregulated by the bacterial challenge in *S. exigua* larvae in fat body and hemocytes (see Supplementary Figure S3). In addition, the immune challenge induced the translocation of Se-DSP1 from the nucleus to extracellular hemocoel. In the naïve larvae of *S. exigua*, Se-DSP1 proteins were localized in the nucleus. However, the bacterial challenge stimulated the translocation of this protein to the cytoplasm and extracellular milieu. This suggests that Se-DSP1 is secreted from cells that recognize an infection-associated signal. In mammals, signals that can induce HMGB1 secretion include pathogen infection, damage, and other stresses such as hypoxia (Andrassy et al., 2008), drug treatment (Ditsworth et al., 2007), and lethal irradiation (Apetoh et al., 2007). Secretion requires the translocation of HMGB1 from the nucleus to the cytoplasm. For such a translocation, a vesicle type of transportation is used for HMGB1 secretion (Wang, 1999; Bianchi et al., 2017). In addition, the exosome appears to be another secretion route for HMGB1 in macrophages (Liu et al., 2006) or platelets (Goetzl et al., 2016). These suggest that, upon immune challenge, Se-DSP1 may translocate from the nucleus to the cytoplasm, where translocated proteins are included in vesicles to be associated with exosomes for secretion to mediate the immune responses of *S. exigua*.

By activating eicosanoid biosynthesis, Se-DSP1 could mediate cellular and humoral immune responses. The nodule formation of hemocytes is a cellular immune response in insects that effectively gets rid of microbial pathogens. Different hemocytes cooperate to make black nodules by enclosing infecting microbes with granulocytes and plasmatocytes. The subsequent melanization by phenoloxidase originates from oenocytoids (Lavine and Strand, 2002; Shrestha and Kim, 2008). The larvae treated with RNAi specifically targeting the *Se-DSP1* expression were significantly impaired in nodule formation. In addition, the RNAi-treated larvae failed to effectively induce AMP gene expression. These findings support that Se-DSP1 has an immune-associated function. Such an immune mediation function of Se-DSP1 might be executed by activating eicosanoid biosynthesis, because the RNAi-treated larvae exhibited a significant reduction in PLA_2_ activities. Furthermore, PLA_2_ catalyzes the committed step for eicosanoid biosynthesis by releasing AA from phospholipids (Kim et al., 2018; Mouchlis and Dennis, 2019). Eicosanoids then mediate cellular and humoral immune responses against various microbial pathogens, such as bacteria, fungi, and viruses (Stanley and Kim, 2020). Thus, the activation of PLA_2_ by Se-DSP1 could upregulate the levels of eicosanoids to mediate immune responses. The role of DSP1 in mediating immune responses *via* eicosanoids was demonstrated in a coleopteran insect, *Tenebrio molitor* (Mollah and Kim, 2021). However, it remains unclear how Se-DSP1 activates PLA_2_ in *S. exigua*. In mammals, extracellular HMGB1 is associated with multiple receptors, such as Toll-like receptors (TLRs), to activate NF-kB (Andersson and Tracey, 2011). Toll immune signaling pathways are conserved in *S. exigua* (Hwang et al., 2013). In toll signaling, a Pelle adaptor is required to activate NF-kB and PLA_2_ in *S. exigua* (Hwang et al., 2013), suggesting that the Se-DSP1 released in the hemolymph can bind to a toll receptor to activate Pelle, a signal component. Pelle then activates PLA_2_, probably by phosphorylation. This suggests that Se-DSP1 may bind to a toll receptor to activate PLA_2_ activity.

Recombinant Se-DSP1 exhibits a relatively high affinity (Kd = 1.28 μM) to SA based on a thermal shift assay. However, binding affinity estimates appear to be different depending on binding assay methods. For example, SA exhibited a binding affinity in the nanomolar range to a reduced form of human HMGB1 by a surface plasmon resonance assay, although other titration method indicated a much lower affinity in the millimolar range (Choi et al., 2015). Choi et al. (2015) have also shown that the binding site of SA is located in boxes A and B of HMGB1, where amino acid residues in these two boxes involved in binding to SA are well-conserved in Se-DSP1, especially in Arg and Lys (see Supplementary Figure S5). Salicylic acid binding to Se-DSP1 had physiological significance, because SA treatment significantly reduced the immune responses of insects. Furthermore, SA has long been used to reduce inflammation (Volt et al., 2009). Aspirin, an SA derivative, has been used for pain relief. It is an anti-inflammatory drug that, by irreversible inhibition *via* the acetylation of COX-2, suppresses PG levels (Ekinci, 2011). Salicylic acid is a metabolite of aspirin in the human body and may increase the anti-inflammatory effect of aspirin (Choi et al., 2015). Immunosuppression was induced by either injecting or feeding SA to the *S. exigua* larvae. As mentioned earlier, ingested SA might bind to extracellular Se-DSP1, but fails to activate PLA_2_ activity upon an immune challenge. Under immunosuppressive conditions, the SA-treated larvae increased their susceptibility to entomopathogenic microbes. Indeed, the SA-treated larvae exhibited increased susceptibility to entomopathogens. This was supported further by assays using larvae fed with endogenous SA *in planta*. Larvae fed with tomato leaves with higher SA levels became more susceptible to entomopathogenic microbes than control larvae that were not fed with such tomato leaves. This suggests that plant hormone SA might have an indirect role in defending phytophagous insects by eliciting tri-trophic interactions. Endogenous plant SA might make insects become more vulnerable to their bacterial pathogens (*B. thuringiensis* or *X. hominickii*), leading to an effective strategy for plants to ward off phytophagous insects. Since the first report showing that SA can induce tobacco mosaic virus resistance in tobacco, it has been found that SA can act as both a phytohormone-mediating plant defense activator and a growth regulator in various plants (Koo et al., 2020). Especially, SA can induce the resistance of plants against different microbial pathogens, such as viruses, bacteria, fungi, and oomycetes. A positive correlation between endogenous levels of SA and resistance responses against biotrophic and hemibiotrophic pathogens has been well-demonstrated (Glazebrook, 2005). This study introduced a novel tri-trophic interaction orchestrated by SA among plant-insect-entomopathogenic microbes. High levels of plant endogenous SA consumed by an herbivore insect (*S. exigua*) caused significant immunosuppression of the insect. Such an effect was mediated by the interaction between SA and Se-DSP1, making the insect vulnerable to entomopathogenic bacteria. Increasing SA content could be a novel crop breeding strategy for insect resistance.

## Data Availability Statement

The datasets presented in this study can be found in online repositories. The names of the repository/repositories and accession number(s) can be found in the article/Supplementary Material.

## Author Contributions

MM and YK carried out the experiment and wrote the manuscript with support from HC, IY, and JL. YK conceived the original idea. YK, IY, HC, and JL supervised the project. All the authors contributed to the article and approved the submitted version.

## Funding

The authors have received funding from the following: a grant (No. 2017R1A2133009815) from the National Research Foundation (NRF) funded by the Ministry of Science, ICT and Future Planning, Republic of Korea.

## Conflict of Interest

The authors declare that the research was conducted in the absence of any commercial or financial relationships that could be construed as a potential conflict of interest.

## Publisher's Note

All claims expressed in this article are solely those of the authors and do not necessarily represent those of their affiliated organizations, or those of the publisher, the editors and the reviewers. Any product that may be evaluated in this article, or claim that may be made by its manufacturer, is not guaranteed or endorsed by the publisher.
