# Supplementary material for: Salicylic Acid, a Plant Hormone, Suppresses Phytophagous Insect Immune Response by Interrupting HMG-Like DSP1
Source: Front Physiol. 2021 Oct 4;12:744272. doi: 10.3389/fphys.2021.744272 (PMC8521015; doi:10.3389/fphys.2021.744272)
Supplement: Supplementary file 2 [file Data_Sheet_2.doc]

**Supplementary Material**

**Table S1.** GenBank accession numbers used for phylogenetic analysis

**Table S2.** Primers used in this study

**Table S3**. Setting values of fluorescence microscope (DM2500, Leica, Wetzlar, Germany) to observe IFA image in **Figure 1**

**Figure S1.** Cross reactivity of a HMGB1 antibody to Se-DSP1: a recombinant Se-DSP1 (‘DSP1’, 1 μg/lane), hemocyte (‘HC’, 5 μg/lane), and fat body (‘FB’, 5 μg/lane) protein extracts of *S. exigua* larvae

**Figure S2.** Molecular characterization of *S. exigua* DSP1 (*Se-DSP1*). **(A)** Phylogenetic analysis of Se-DSP1 performed with MEGA6 program. Bootstrapping values were obtained with 1,000 repetitions to support branching and clustering. Amino acid sequences were retrieved from GenBank with accession numbers shown in Table S1. **(B)** Domain analysis of *Se-DSP1*. Domains were predicted using Prosite (https://prosite.expasy.org/) and SMART protein (http://smart.embl-heidelberg.de/).

**Figure S3.** Expression profiles of *Se-DSP1*. (A) Expression pattern at different developmental stages: egg, first to fifth instar larvae (‘L1-L5’), pupa, and adult. (B) Expression patterns in indicated tissues of L5 larvae: hemocyte (‘HC’), fat body (‘FB’), midgut (‘GT’) and epidermis (‘EPD’). (C) Inducible expression of *Se-DSP1* in HC and FB of L5 larvae at different time intervals after bacterial infection. Bacterial challenge was performed by injecting 1.5 × 10^5^ cells of *E. coli* to each larva. For control, larvae were injected with sterile water. Up-regulated expression levels were calculated as fold change (‘FC’) of expression levels between naïve and bacteria-challenged larvae. A ribosomal gene, RL32, was used as a reference gene. Each treatment was replicated three times with independent tissue preparations. Different letters above the bar indicate significant differences among means at type I error = 0.05 (LSD test).

**Figure S4.** LC-MS/MS analysis of a partial Se-DSP1

**Figure S5.** Conserved residues of Se-DSP1 binding to salicylic acid (SA)

**Table S1.** GenBank accession numbers used for phylogenetic analysis.

| **Species** | **Gene** | **Accession number** | |
| --- | --- | --- | --- |
| *Raphanus sativus* | HMGB1 | | XP_018433380.1 |
| *Arabidopsis thaliana* | HMGB1 | | AEE78856.1 |
| *Gossypium arboreum* | HMGB1 | | KHG02391.1 |
| *Citrus sinensis* | HMGB1 | | XP_006481020.1 |
| *Nicotiana attenuata* | HMGB1 | | OIT20918.1 |
| *Dendrobium catenatum* | HMGB1 | | PKU75600.1 |
| *Oryza sativa* ssp. *indica* | HMGB1 | | AAP21609.1 |
| *Plasmodium ovale* | HMGB1 | | SCP05959.1 |
| *Plasmodium malariae* | HMGB1 | | SBT71922.1 |
| *Strongyloides ratti* | DSP1 | | XP_024506421.1 |
| *Echinococcus granulosus* | DSP1 | | XP_024348839.1 |
| *Danio rerio* | HMGB1 | | AAI65307.1 |
| *Labeo rohita* | HMGB1 | | ARX80203.1 |
| *Chrysemys picta bellii* | HMGB1 | | XP_005286694.1 |
| *Pogona vitticeps* | HMGB1 | | XP_020644230.1 |
| *Ophiophagus hannah* | HMGB1 | | ETE66153.1 |
| *Homo sapiens* | HMGB1 | | CAG33144.1 |
| *Bos indicus* | HMGB1 | | AIA24584.1 |
| *Mus musculus* | HMGB1 | | AAI10668.1 |
| *Rhinolophus ferrumequinum* | HMGB1 | | ACC62098.1 |
| *Brachionus plicatilis* | DSP1 | | RNA19426.1 |
| *Penaeus vannamei* | DSP1 | | XP_027210949.1 |
| *Trichonephila* (*Nephila*) *clavipes* | DSP1 | | PRD29151.1 |
| *Tetranychus urticae* | DSP1 | | XP_015795363.1 |
| *Tribolium castaneum* | DSP1 | | XP_973934.2 |
| *Leptinotarsa decemlineata* | DSP1 | | XP_023011517.1 |
| *Spodoptera exigua* | DSP1 | | MK 737894 |
| *Helicoverpa armigera* | DSP1 | | XP_021183962.1 |
| *Bombyx mori* | DSP1 | | XP_012547112.1 |
| *Galleria mellonella* | DSP1 | | XP_026748165.1 |
| *Plutella xylostella* | DSP1 | | XP_011560328.1 |
| *Ctenocephalides felis* | DSP1 | | XP_026466903.1 |
| *Drosophila melanogaster* | DSP1 | | AAN09395.1 |
| *Anopheles darlingi* | DSP1 | | ETN66707.1 |
| *Myzus persicae* | DSP1 | | XP_022178944.1 |
| *Rhopalosiphum maidis* | DSP1 | | XP_026806372.1 |
| *Nasonia vitripennis* | DSP1 | | XP_016839160.1 |
| *Habropoda laboriosa* | DSP1 | | KOC71292.1 |
| *Azumapecten farreri* | HMGB1 | | AFN27605.1 |
| *Crassostrea gigas* | HMGB1 | | AGH28093.1 |
| *Saccostrea kegaki* | HMGB1 | | BAG55013.1 |
| *Rhizopus azygosporus* | HMGB1 | | RCI00565.1 |

**Table S2. Primers used in this study**

| **Genes** | **Primer sequences** | **Purpose** |
| --- | --- | --- |
| *Se-DSP1* | 5´-CAAGCTGCATTACAACGGCA-3´  5´-CAGGGGACCCTTCTTGTACG-3´ | RT-qPCR |
|  | 5´-ATGGGGGATCGGGGCGCGAC-3´  5´-CTTGTACTCCTCCTCGGCGTCG-3´ | ORF cloning |
| *Apolipophorin III* | 5´-AGTGTCGCCAAGTTGTTCGTG-3´  5´-CTCCTGCGCGGTGTTCTGCA-3´ | RT-qPCR |
| *Attacin* | 5´-GCTTTCCTCTCCAGGAATATG-3´  5´-CCTTAGAGTAAATCCAGTGG-3´ | RT-qPCR |
| *Cecropin* | 5´-ATCGTTTAGCTTCGTGTTCGC-3´  5´-CTTTCTTTTACCACACGGTTG-3´ | RT-qPCR |
| *Defensin I* | 5´-ATGGGTGTTAAGGTAATAAATGTG-3´  5´-GCAACTACATGTATGACTAACGC-3´ | RT-qPCR |
| *Gallerimycin* | 5´-TCAGTCATGAAAGCTTGCGTA-3´  5´-TCGCACACATTGGCATCCATTC-3´ | RT-qPCR |
| *Transferrin I* | 5´-GTCCCTCTCTGTCCTGAAGG-3´  5´-CAGAAACACGAAGAAAGATGG-3´ | RT-qPCR |

**Table S3**. Setting values of fluorescence microscope (DM2500, Leica, Wetzlar, Germany) to observe IFA image in **Figure 3**

| **Tissues** | **Treatment** | **F-actin** | **Nucleus** | **Se-DSP1** |
| --- | --- | --- | --- | --- |
| **Hemocyte** | Naive | Magnification at 400; Color Mode; Exposure-16.50; Contrast-1.0 | Magnification at 400; Color Mode; Exposure-180; Contrast-1.20 | Magnification at 400; Color Mode; Exposure-25.50; Contrast-1.50 |
|  | Immune | Magnification at 400; Color Mode; Exposure-15.50; Contrast-1.0 | Magnification at 400; Color Mode; Exposure-180; Contrast-1.20 | Magnification at 400; Color Mode; Exposure-26.50; Contrast-1.50 |
| **Fat body** | Naive | Magnification at 400; Color Mode; Exposure-15.50; Contrast-1.0 | Magnification at 400; Color Mode; Exposure-180; Contrast-1.20 | Magnification at 400; Color Mode; Exposure-27.50; Contrast-1.30 |
|  | Immune | Magnification at 400; Color Mode; Exposure-15.50; Contrast-1.0 | Magnification at 400; Color Mode; Exposure-190; Contrast-1.20 | Magnification at 400; Color Mode; Exposure-27.50; Contrast-1.30 |


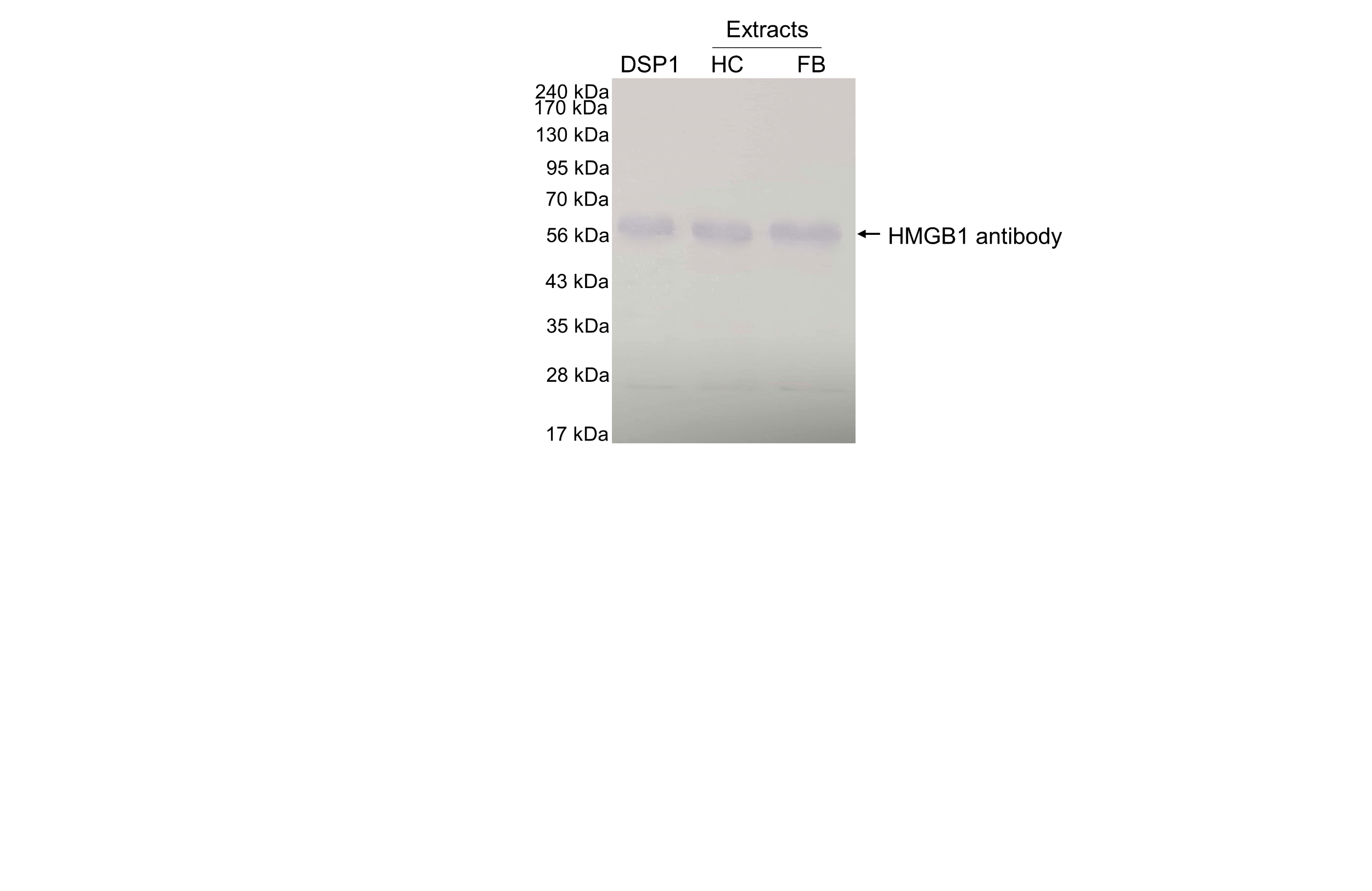


**Figure S1**


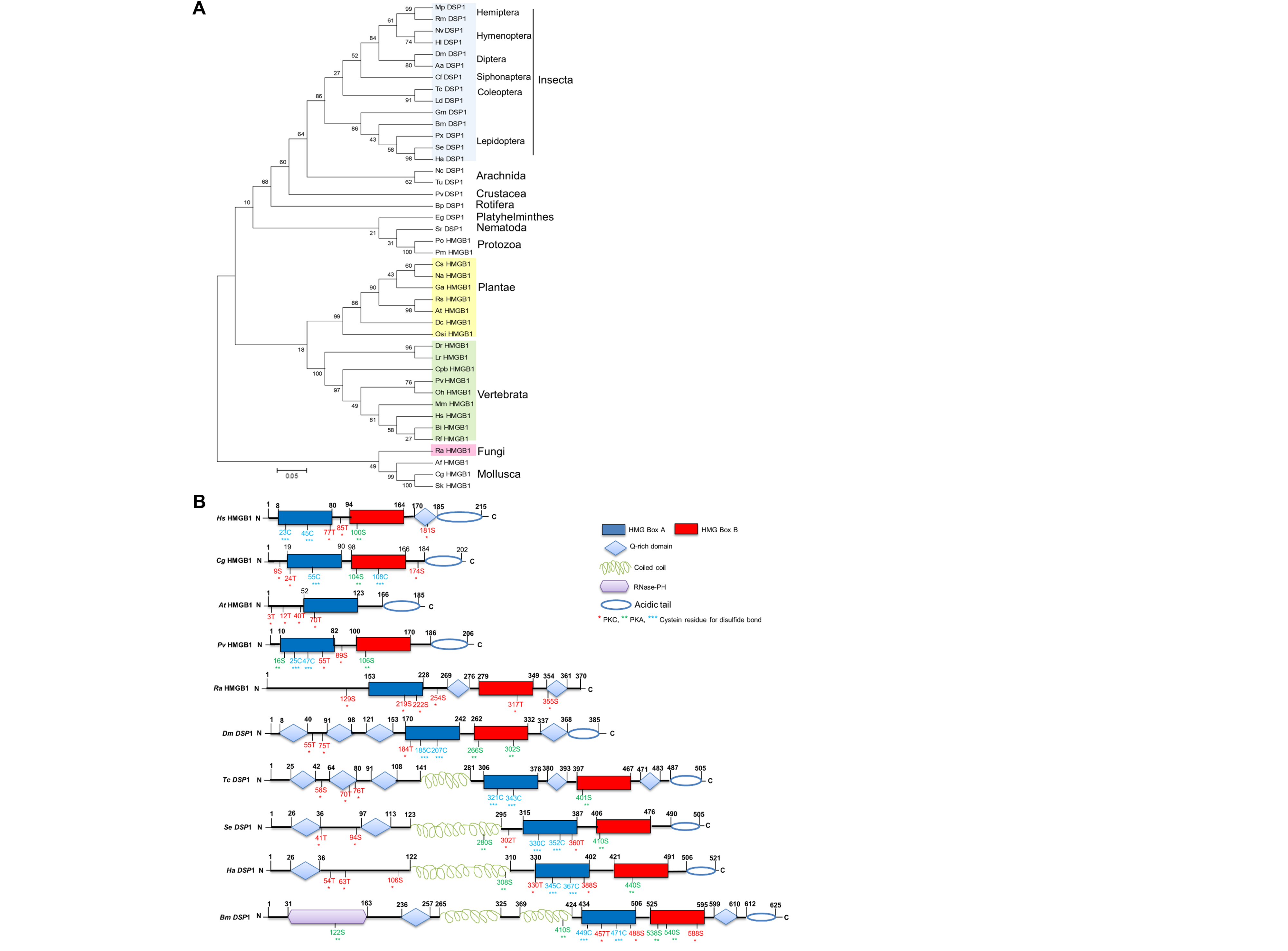


**Figure S2**


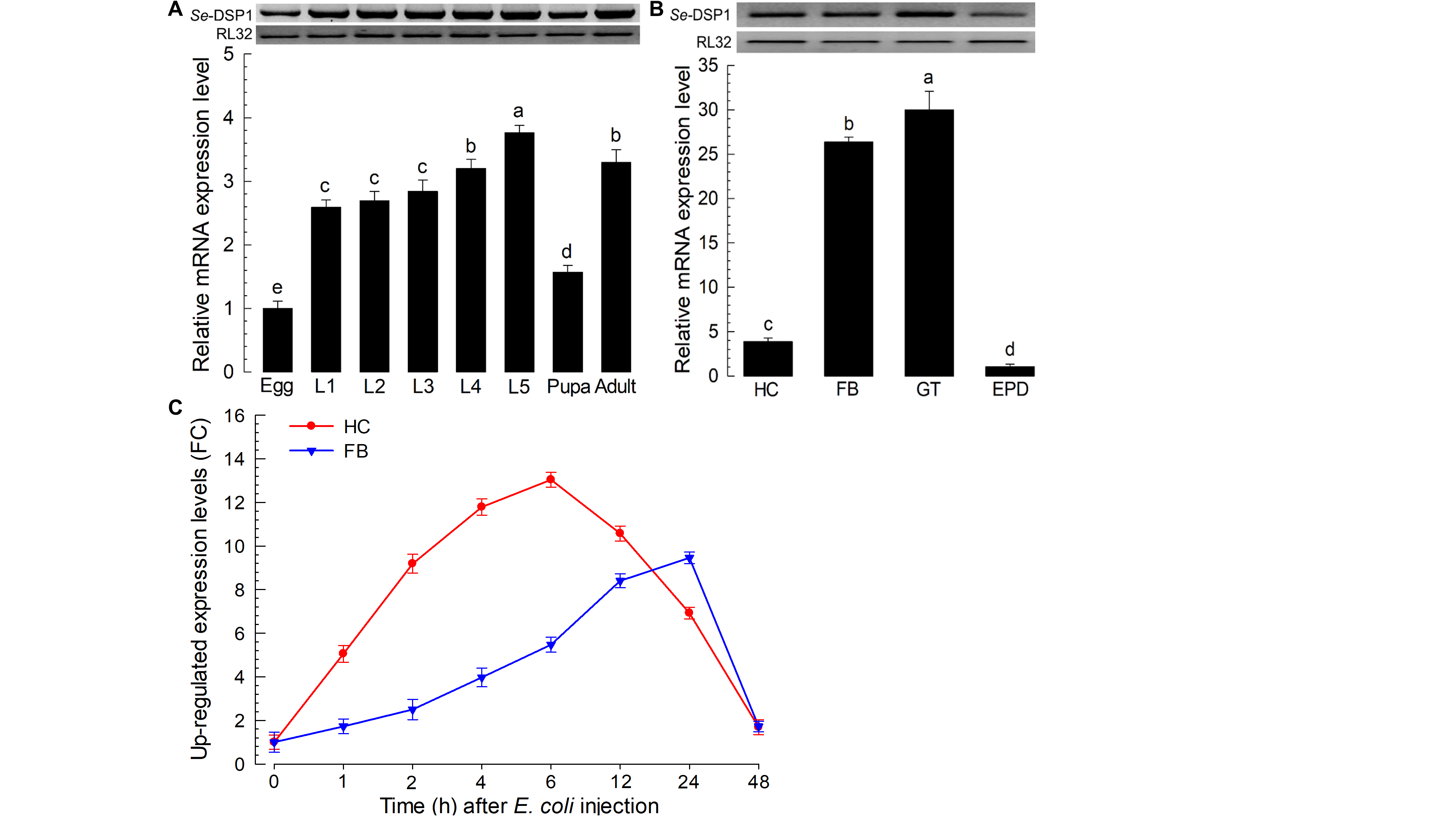


**Figure S3**


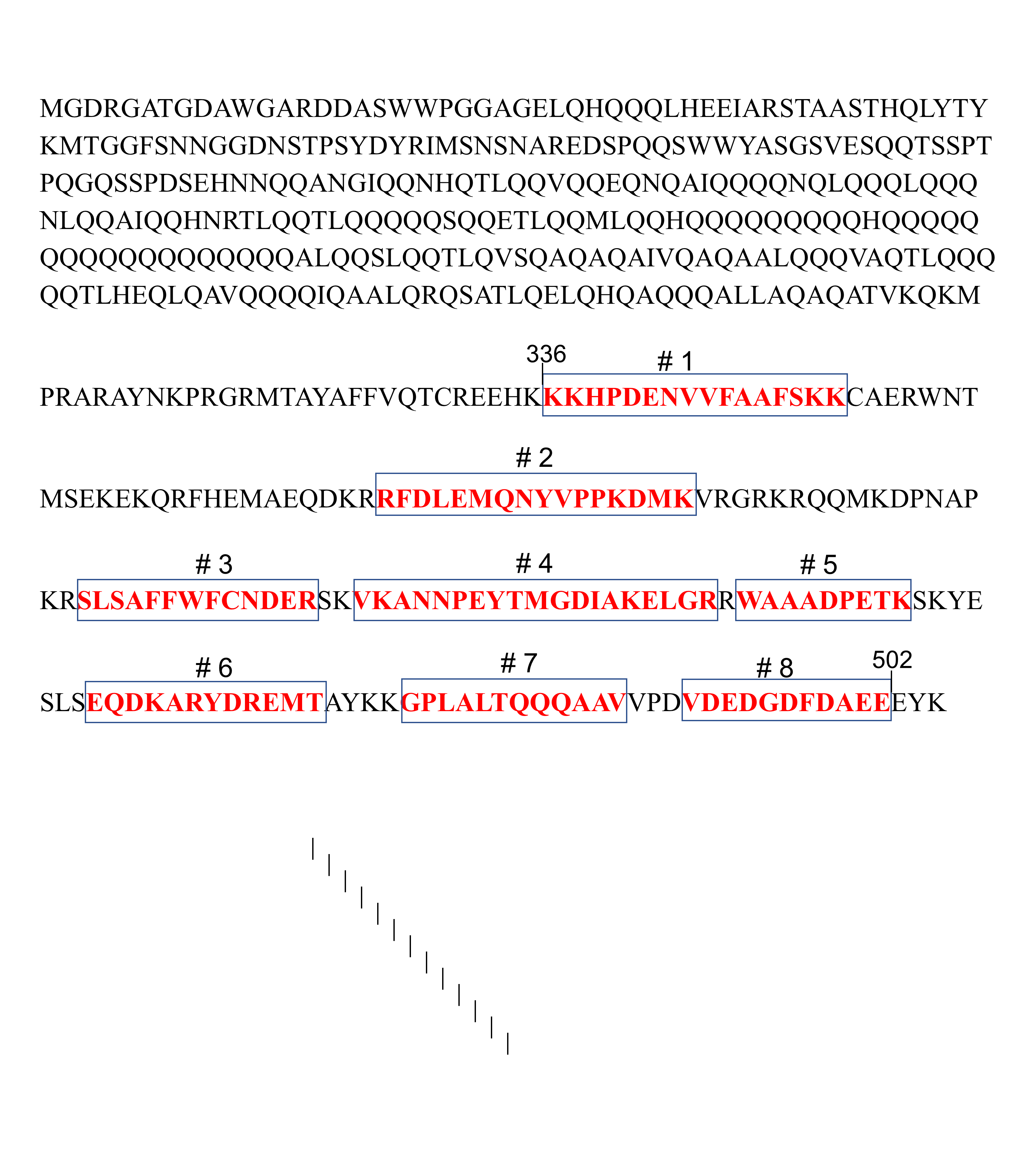


| LC-MS/MS Fragment | Number of hits | Amino Acid (AA)  Sequence | Accession  number | Predicted gene | Match Score | |
| --- | --- | --- | --- | --- | --- | --- |
|  |  |  |  |  | Query  cover | Identity  (%) |
| # 1 | 3 | KKHPDENVVFAAFSKK | QFU47757.1 | DSP1  (*Spodoptera exigua)* | 100 | 100 |
| # 2 | 6 | RFDLEMQNYVPPKDMK | QFU47757.1 | DSP1  (*Spodoptera exigua)* | 100 | 100 |
| # 3 | 4 | SLSAFFWFCNDER | QFU47757.1 | DSP1  (*Spodoptera exigua)* | 100 | 100 |
| # 4 | 8 | VKANNPEYTMGDIAKELGR | QFU47757.1 | DSP1  (*Spodoptera exigua)* | 100 | 100 |
| # 5 | 6 | WAAADPETK | QFU47757.1 | DSP1  (*Spodoptera exigua)* | 100 | 100 |
| # 6 | 7 | EQDKARYDREMT | QFU47757.1 | DSP1  (*Spodoptera exigua)* | 100 | 100 |
| # 7 | 9 | GPLALTQQQAAV | QFU47757.1 | DSP1  (*Spodoptera exigua)* | 100 | 100 |
| # 8 | 5 | VDEDGDFDAEE | QFU47757.1 | DSP1  (*Spodoptera exigua)* | 100 | 100 |

**Figure S4**


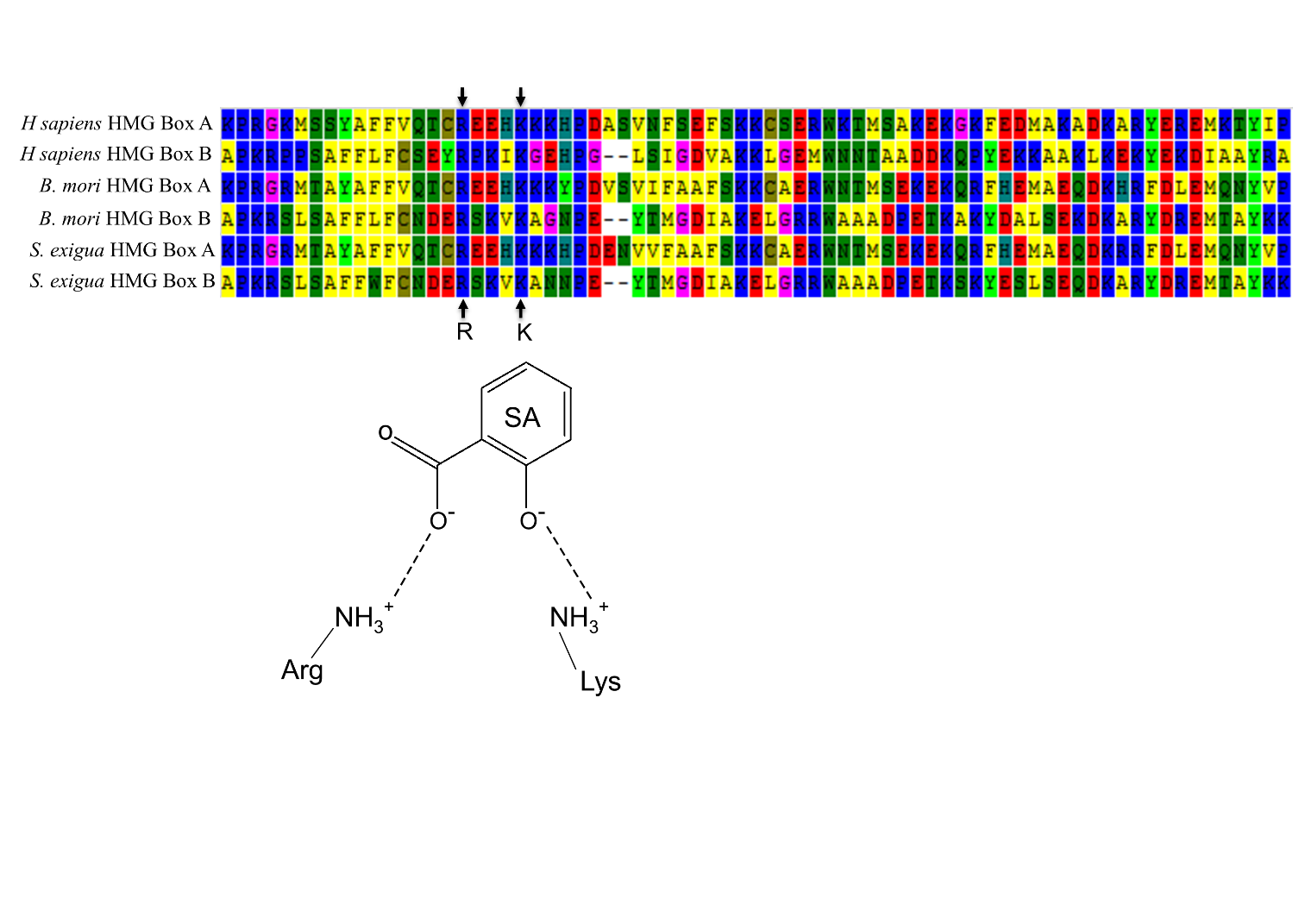


**Figure S5**
